# Supplementary material for: Accurate quantification of circular RNAs identifies extensive circular isoform switching events
Source: Nat Commun. 2020 Jan 3;11:90. doi: 10.1038/s41467-019-13840-9 (PMC6941955; doi:10.1038/s41467-019-13840-9)
Supplement: Supplementary file 1 — Supplementary Information [file 41467_2019_13840_MOESM1_ESM.pdf]

Supplementary Information

Accurate quantification of circular RNAs identifies extensive circular isoform switching events

Zhang et al.

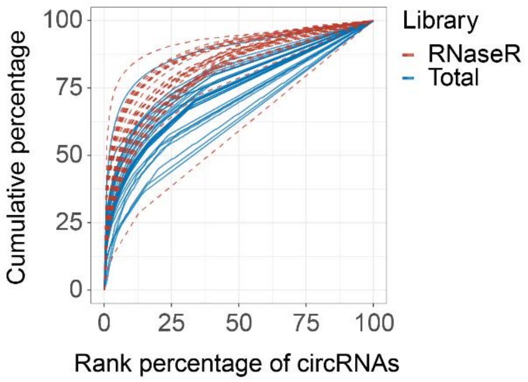

Supplementary Figure 1. Cumulative percentage distribution of circRNA expression after RNase R treatment. The horizontal axis represents the percentage of rank based on the expression level in all circRNAs.

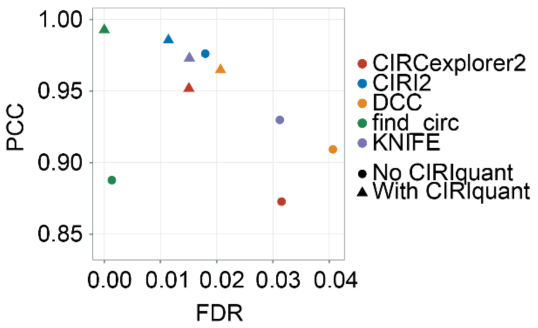

Supplementary Figure 2. Assessment of computational tools on circRNA detection and quantification. The Pearson correlation coefficient and false discovery rate of five algorithms before and after CIRIquant correction using the simulated dataset. Triangular and circular points indicate the results with and without CIRIquant correction, respectively.

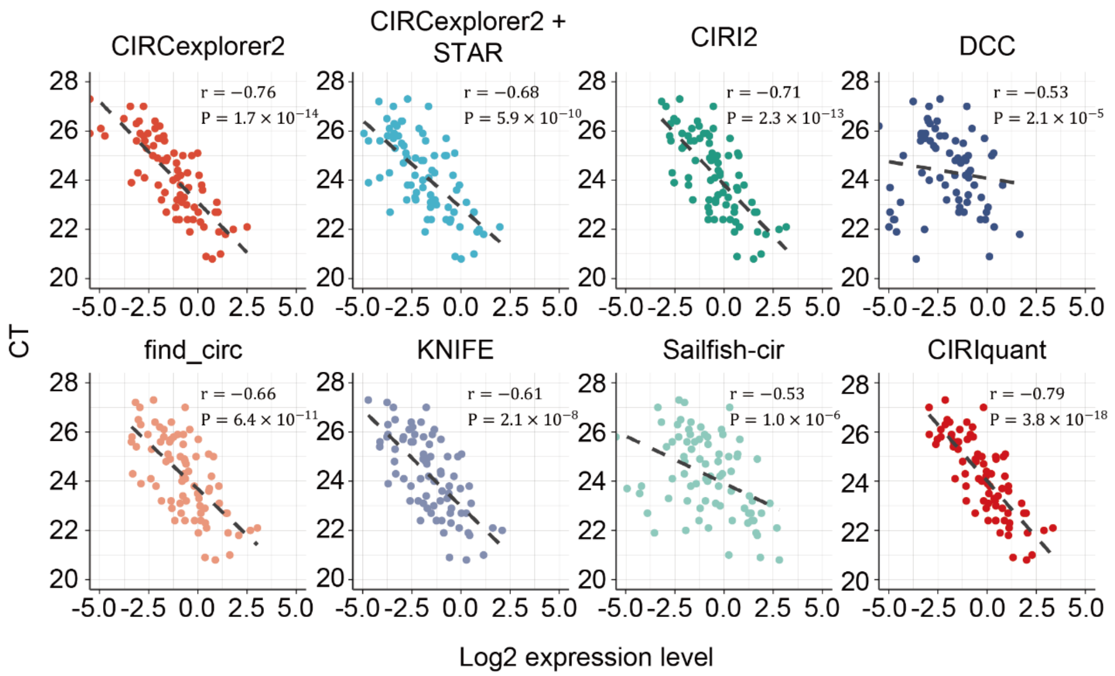

Supplementary Figure 3. Correlation between qRT-PCR and circRNA quantification results. Each dot represents one circRNA transcript. X-axis represents the log2 normalized expression value of circRNAs. Y-axis represents the CT value in qRT-PCR experiment. CIRIquant shows better performance than all other tools on both correlation coefficient (r) and p value. For Sailfish-cir, the TPM was used to measure the circRNA expression level, while CPM (counts per million, calculated as #BSJ / mapped reads * 1000000) was used for the other BSJ-based tools.

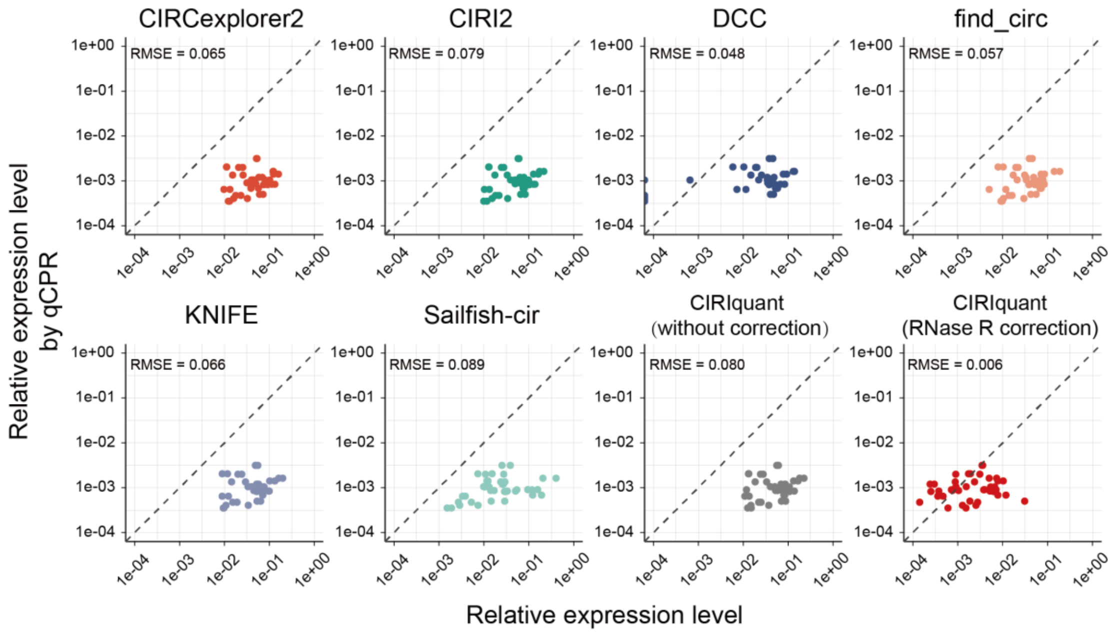

Supplementary Figure 4. Quantitative reverse transcription PCR on five circRNAs in HeLa cells demonstrates the efficiency of CIRIquant on RNase R treatment correction. X- and y- axis represent the relative fold of circRNA expression levels to GAPDH. Five circRNAs were randomly selected, and qRT-PCR were performed in control / MBKD / TrKD / PTKD libraries. For each sample, all six tools were applied to calculate the expression value of five circRNAs in two RNase R replicates, respectively. Root-mean-squared error (RMSE) between the predicted expression value in RiboMinus/RNase R libraries and the experimentally validated value in RiboMinus datasets were used for performance evaluation. For CIRIquant, we firstly calculated the CPM using RiboMinus/RNase R treated sample only and performed RNase R correction using both RiboMinus and RiboMinus/RNase R data. After the correction step, the RMSE of CIRIquant is significantly smaller, which indicate the effective correction of bias induced by RNase R treatment.

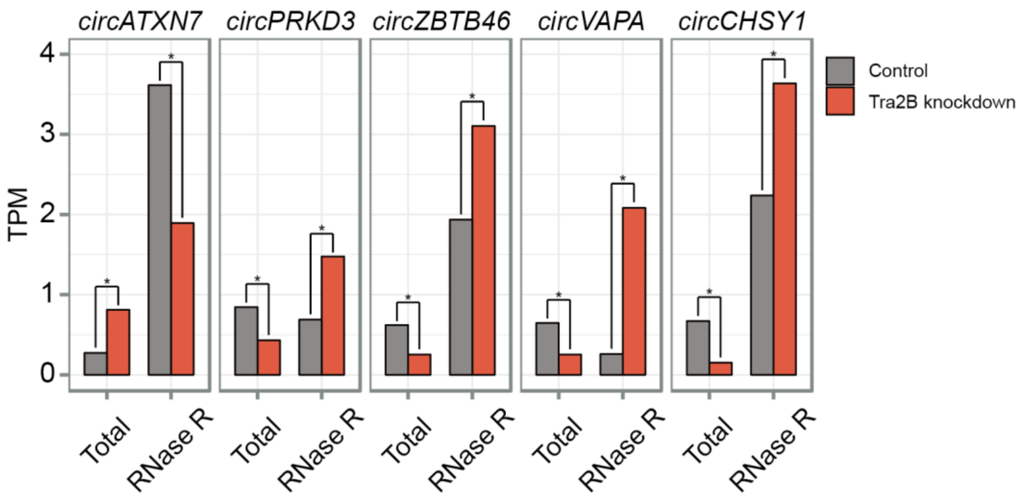

Supplementary Figure 5. Five examples illustrate the contradictory expression changes after TRA2B knockdown between RiboMinus and RiboMinus/RNase R samples. CPM of circRNAs in control set (grey) and TRA2B knockdown data (red) were calculated in RiboMinus and RiboMinus/RNase R libraries separately.

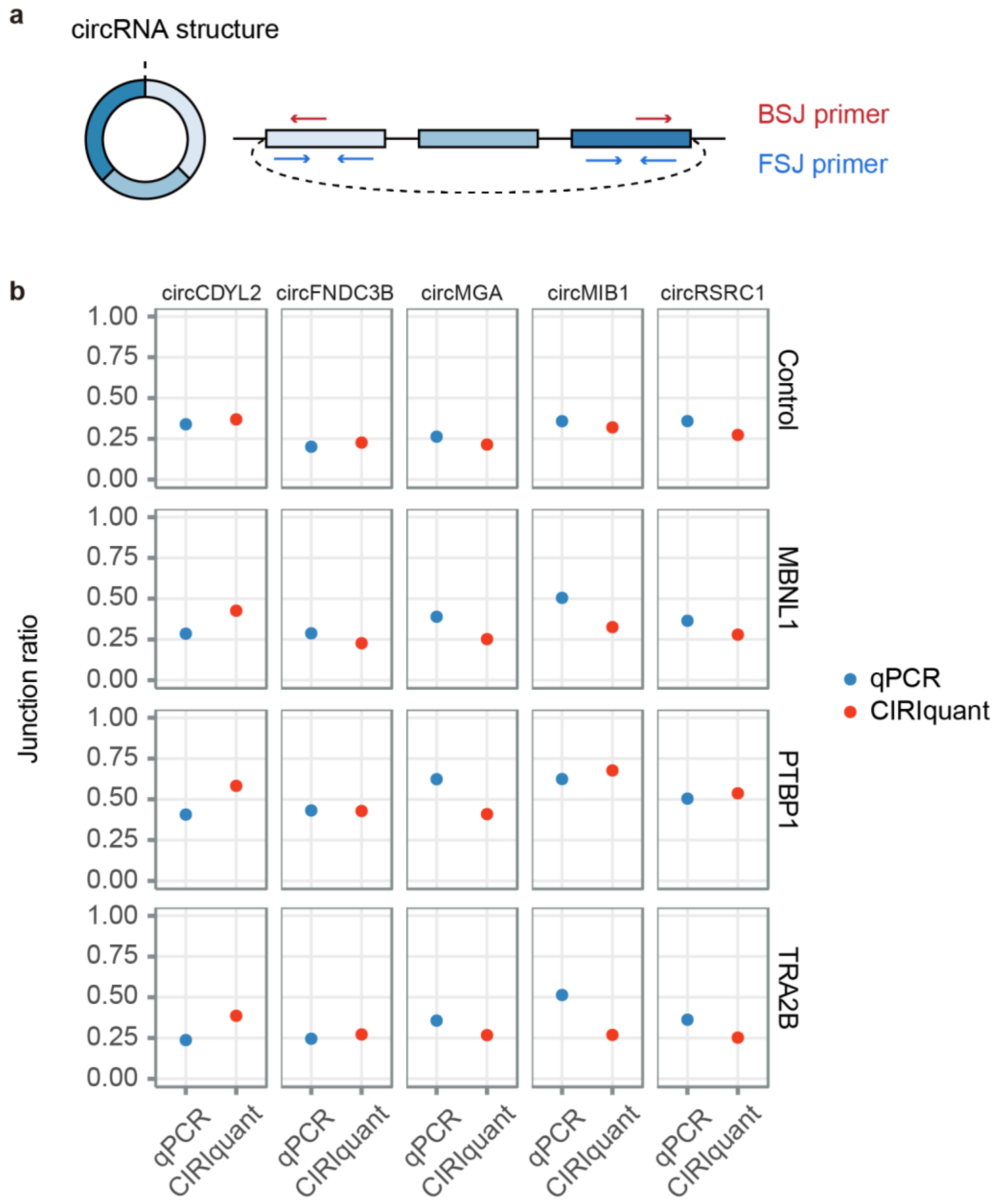

Supplementary Figure 6. RT-qPCR validation of circRNA junction ratios. *(A)* The design of outward and inward primers for detection of BSJ and 5'/3' forward spliced exons. *(B)* Y-axis represents the junction ratio of five circRNAs in four libraries by qPCR and CIRIquant. The junction ratio of circRNAs is determined from the qPCR results by dividing the relative expression of BSJ by the sum of FSJ expression values.

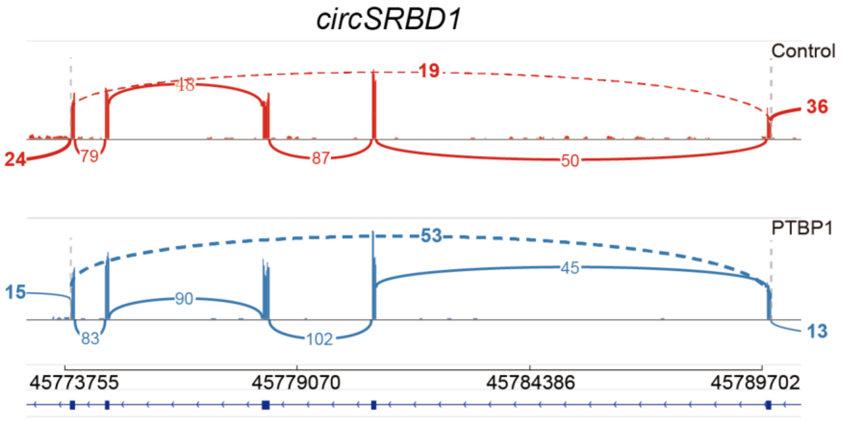

Supplementary Figure 7. Sashimi plot shows the change of BSJ reads number and junction ratio in circSRBD1. The dashed line represents the back-spliced junction of circSRBD1, and the number on the curved line indicates the number of reads supporting the BSJ or FSJ signal.

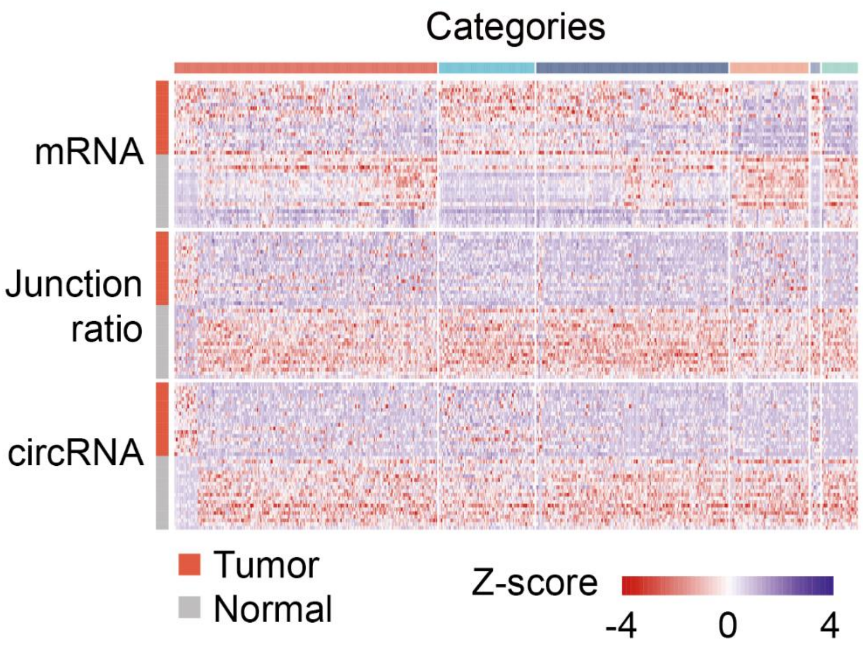

Supplementary Figure 8. Hierarchical clustering Heatmap of differentially expressed circRNAs (DE- and DS-) and their host genes. All These circRNAs are divided into 6 categories based on the overlap information in Venn diagram (Figure 6B), and hierarchical clustered inside each group. Data were scaled to columns using Z-score and a distinct trend of DE- and DS- circRNA was observed.

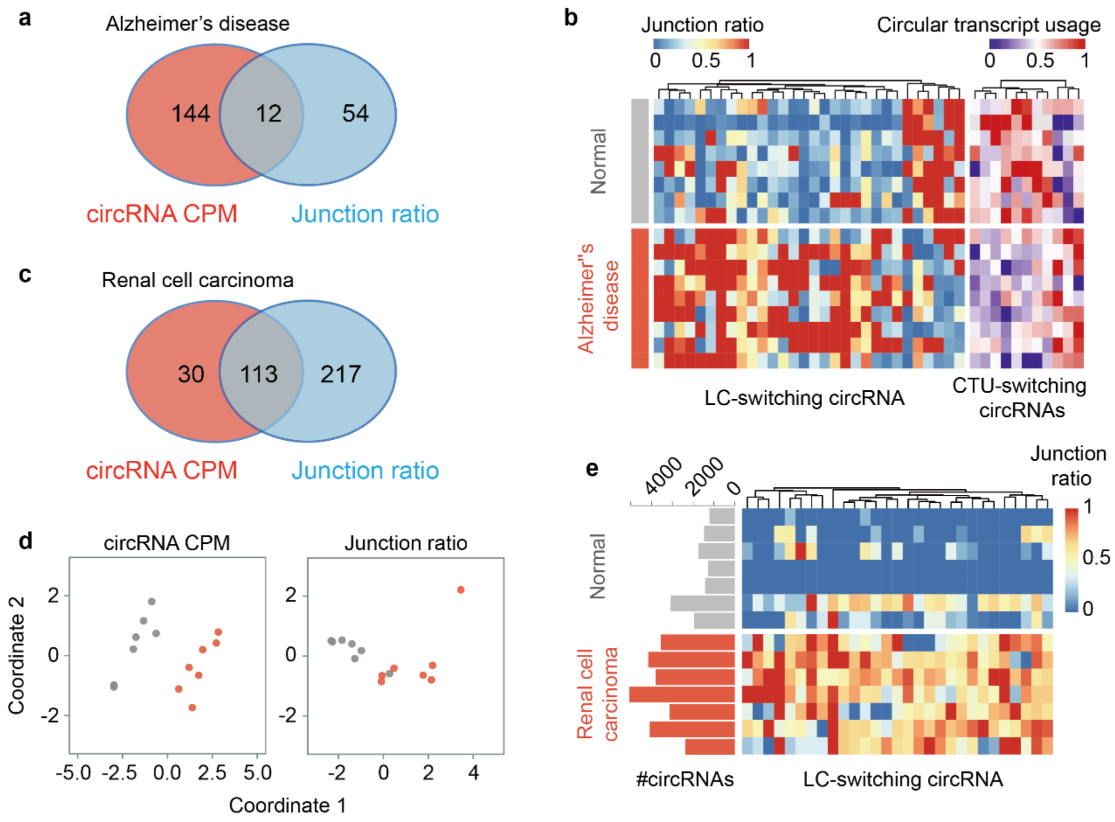

Supplementary Figure 9. LC-switching and CTU-switching events in Alzheimer's Disease and Renal cell carcinoma datasets. Additional RNA-seq data were downloaded from two previous studies (PRJNA232669 and PRJNA428447). The first dataset contains 7 normal human brain samples and 8 advanced Alzheimer's disease brain samples; the second dataset composes 7 pairs of carcinoma/normal tissues from patients with renal cell carcinoma. (A) Overlap of DE-circRNAs and DS-circRNAs in Alzheimer's Disease patients compared to normal brain samples. (B) Hierarchical clustering of 28 LC-switching and 11 CTU-switching circRNAs in Alzheimer's Disease data. The junction ratio and circular transcript usage of these circRNAs were plotted respectively. (C) Overlap of DE-circRNAs and DS-circRNAs in Renal Cell Carcinoma (RCC) samples compared to normal kidney samples. (D) Multi-dimension analysis using circRNA expression values (CPM) and junction ratios. The RCC samples and normal controls were clearly distinguished from each other. (E) Hierarchical clustering of 29 LC-switching in RCC data. The bar plot showed the amount of circRNAs detected in each sample.

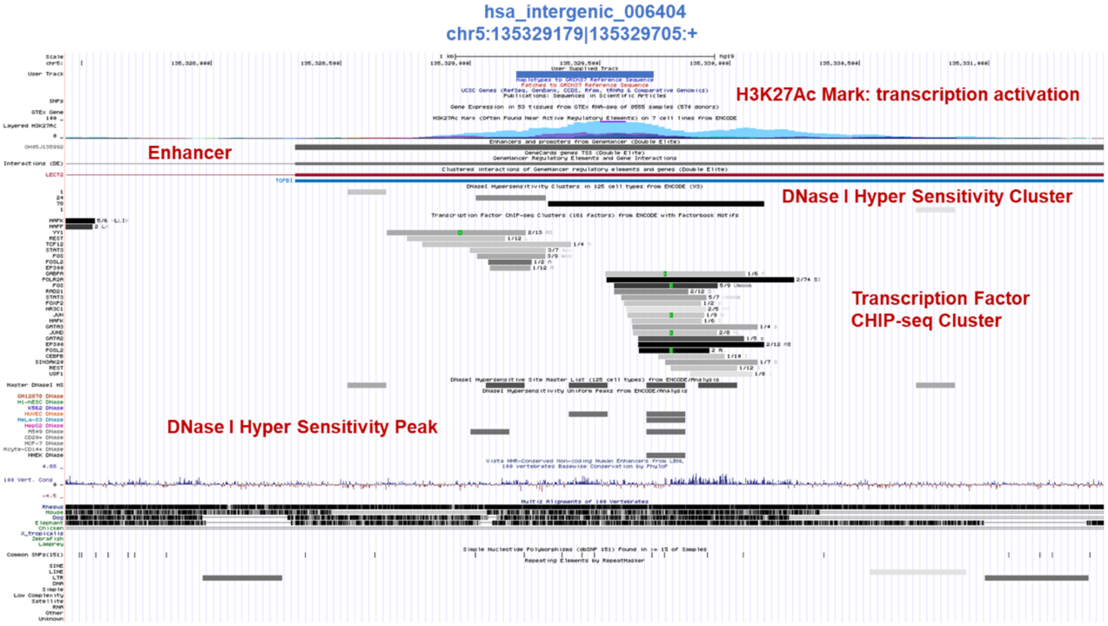

Supplementary Figure 10 | Functional elements in the flanking region of hsa_intergenic_006404.

The blue rectangle shows the coordinate of back-splice junction site of hsa_intergenic_006404. The hsa_intergenic_006404 was derived from an enhancer region, and DNase I hypersensitivity peak along with H3K27Ac mark was observed inside the BSJ and also in its flanking region. The transcription factor CHIP-seq track also exhibits enriched clusters inside the BSJ.

Supplementary Table 1. Public RNA-seq data

Sample_Name	Experiment	LibraryLayout	MBases	MBytes	Organism	Library_Name	Treatment	Reference
GSM1558150	SRX795207	SINGLE	1176	699	Caenorhabditis elegans	embryo	none	10.1016/j.celrep.2014.12.019
GSM1558151	SRX795208	SINGLE	2723	1593	Caenorhabditis elegans	embryo	RNaseR	
GSM1558152	SRX795209	SINGLE	2036	1193	Caenorhabditis elegans	L1-L4	none	
GSM1558153	SRX795210	SINGLE	2532	1486	Caenorhabditis elegans	L1-L4	RNaseR	
GSM1558154	SRX795211	SINGLE	1514	895	Caenorhabditis elegans	L1-L4	none	
GSM1558155	SRX795212	SINGLE	2416	1414	Caenorhabditis elegans	L1-L4	RNaseR	
GSM1558156	SRX795213	SINGLE	1756	1040	Caenorhabditis elegans	L1-L4	none	
GSM1558157	SRX795214	SINGLE	2487	1464	Caenorhabditis elegans	L1-L4	RNaseR	
GSM1558158	SRX795215	SINGLE	1823	1068	Caenorhabditis elegans	L1-L4	none	
GSM1558159	SRX795216	SINGLE	2155	1268	Caenorhabditis elegans	L1-L4	RNaseR	
GSM1558160	SRX795217	SINGLE	1626	962	Caenorhabditis elegans	Young Adult	none	
GSM1558161	SRX795218	SINGLE	2589	1523	Caenorhabditis elegans	Young Adult	RNaseR	
GSM1347830	SRX488409	SINGLE	1885	1145	Drosophila melanogaster	wild type Canton-S	none	
GSM1347831	SRX488410	SINGLE	2419	1467	Drosophila melanogaster	wild type Canton-S	none	
GSM1347832	SRX488411	SINGLE	1872	1132	Drosophila melanogaster	C4 mutation Canton-S	none	
GSM1347833	SRX488412	SINGLE	1641	994	Drosophila melanogaster	C4 mutation Canton-S	none	
GSM1347834	SRX488413	SINGLE	1687	1020	Drosophila melanogaster	wild type Canton-S	RNaseR	
GSM1347835	SRX488414	SINGLE	1623	981	Drosophila melanogaster	wild type Canton-S	RNaseR	
GSM1347836	SRX488415	SINGLE	1795	1089	Drosophila melanogaster	C4 mutation Canton-S	RNaseR	
GSM1347837	SRX488416	SINGLE	1607	972	Drosophila melanogaster	C4 mutation Canton-S	RNaseR	
GSM1347838	SRX488417	SINGLE	1463	906	Drosophila melanogaster	wild type Canton-S	none	
GSM1347839	SRX488418	SINGLE	2317	1434	Drosophila melanogaster	wild type Canton-S	none	
GSM1347840	SRX488419	SINGLE	1583	977	Drosophila melanogaster	C4 mutation Canton-S	none	
GSM1347841	SRX488420	SINGLE	2268	1404	Drosophila melanogaster	C4 mutation Canton-S	none	
GSM1347842	SRX488421	SINGLE	1830	1127	Drosophila melanogaster	wild type Canton-S	RNaseR	
GSM1347843	SRX488422	SINGLE	1773	1094	Drosophila melanogaster	wild type Canton-S	RNaseR	

GSM1347844	SRX488423	SINGLE	1972	1214	Drosophila melanogaster	C4 mutation Canton-S	RNaseR	
GSM1347845	SRX488424	SINGLE	1972	1218	Drosophila melanogaster	C4 mutation Canton-S	RNaseR	
GSM1347846	SRX488425	SINGLE	4292	2717	Drosophila melanogaster	<not provided>	none	
GSM1347847	SRX488426	SINGLE	4488	2852	Drosophila melanogaster	<not provided>	RNaseR	
OVCAR3_RNaseR	SRX852975	PAIRED	12780	6247	Homo sapiens	OVCAR3_RNaseR	RNaseR	10.5339/qfarc.2014.HBOP0974
OVCAR3	SRX852524	PAIRED	12328	5916	Homo sapiens	OVCAR3	none	
SKOV3_RNaseR	SRX857037	PAIRED	10172	4823	Homo sapiens	SKOV3_RNaseR	RNaseR	
SKOV3	SRX857038	PAIRED	11427	5506	Homo sapiens	SKOV3	none	
Biological Replicate 1	SRX122237	PAIRED	29955	18978	Homo sapiens	Rep1control	none	10.1261/rna.035667.112
Biological Replicate 1	SRX122238	PAIRED	30194	19954	Homo sapiens	Rep1RNAseR	RNaseR	
Biological Replicate 2	SRX122239	PAIRED	39360	27697	Homo sapiens	Rep2control	none	
Biological Replicate 2	SRX122240	PAIRED	38132	26556	Homo sapiens	Rep2RNAseR	RNaseR	
GSM1964866	SRX1466118	SINGLE	8361	4588	Homo sapiens	PA1	none	10.1101/gr.202895.115
GSM1964867	SRX1466119	SINGLE	6669	4193	Homo sapiens	PA1	RNaseR	
RnaSeq_HeLa_cell_RNaseR	SRX749316	PAIRED	5229	3306	Homo sapiens	Sample_2016_RIBO	none	10.1038/ncomms12060
RnaSeq_HeLa_cell_RNaseR	SRX749241	PAIRED	10123	6548	Homo sapiens	Sample_4031_RIQ	RNaseR	
HEK293 Cell Line	SRX1744886	PAIRED	9317	5882	Homo sapiens	RnaSeq_HEK293	none	
HEK293 Cell Line	SRX1744886	PAIRED	12692	7986	Homo sapiens	RnaSeq_HEK293	RNaseR	
GSM1480601	SRX682271	SINGLE	3383	2161	Mus musculus	R1	none	10.1016/j.cell.2014.09.001
GSM2039387	SRX1530982	SINGLE	5969	3858	Mus musculus	R1	RNaseR	
total_RNA_mouse_brain	SRX1165561	PAIRED	6001	4265	Mus musculus	8weeks_brain	none	https://trace.ddbj.nig.ac.jp/DRASearch/submission?acc=SRA291626
total_RNA_mouse_brain	SRX1175091	PAIRED	4099	2915	Mus musculus	8weeks_brain	RNaseR	

Supplementary Table 2. Gene Ontology enrichment analysis of DE&DS-circRNAs and DE-genes in PTBP1 knockdown data

**DE- & DS-circRNAs**				
Term	Overlap	P-value	Adjusted P-value	Genes
regulation of vascular smooth muscle cell proliferation (GO:1904705)	3/26	0.000101	0.025594	JUN;TPM1;FGF2
positive regulation of nitric oxide biosynthetic process (GO:0045429)	3/26	0.000101	0.025594	CD36;PTGS2;TLR4
positive regulation of nitric oxide metabolic process (GO:1904407)	3/26	0.000101	0.025594	CD36;PTGS2;TLR4
positive regulation of reactive oxygen species biosynthetic process (GO:1903428)	3/31	0.000172	0.031664	CD36;PTGS2;TLR4
regulation of nitric oxide biosynthetic process (GO:0045428)	3/33	0.000208	0.031664	CD36;PTGS2;TLR4
interleukin-1 beta production (GO:0032611)	2/10	0.000534	0.049587	CD36;TLR4
interleukin-1 beta secretion (GO:0050702)	2/9	0.000428	0.049587	CD36;TLR4
interleukin-1 secretion (GO:0050701)	2/10	0.000534	0.049587	CD36;TLR4
regulation of NLRP3 inflammasome complex assembly (GO:1900225)	2/11	0.000651	0.049587	CD36;TLR4
regulation of cell motility (GO:2000145)	4/110	0.000601	0.049587	CDK6;SERPINE2;GATA3;RND2
**DE-genes**				
Term	Overlap	P-value	Adjusted P-value	Genes
protein phosphorylation (GO:0006468)	27/471	4.2E-06	0.00852	GSK3B;CTBP1;STK4;NLK;AURKA;IGF1R;MAPK9;PPP4R1;ERBB2;RIPK1;RICTOR;ERC1;MAPK6;MAP3K5;SMAD2;CSNK1G3;CAMK1D;DCLK2;LMTK2;VRK1;CDC42BPA;HIPK3;PTK2;WNK1;FAM20B;PKN2;FGFR2
DNA repair (GO:0006281)	19/289	1.75E-05	0.011851	SETD2;FANCL;HUWE1;FANCC;CHD1L;FANCB;PDS5A;RAD23B;BACH1;BABAM1;POLA1;BRIP1;NIPBL;UIMC1;POLI;ASCC3;DNA2;ERCC6;RAD18
peptidyl-serine phosphorylation (GO:0018105)	13/146	1.67E-05	0.011851	GSK3B;CSNK1G3;CAMK1D;DCLK2;LMTK2;VRK1;STK4;NLK;HIPK3;MAPK9;PKN2;RIPK1;RICTOR
Golgi vesicle budding (GO:0048194)	4/10	3.99E-05	0.020235	GOLPH3;VAPA;VAPB;SEC31A

Supplementary Table 3. LC-switching circRNAs in three splicing factor knockdown datasets

circRNA_ID	Sample	Ctrl Junction Ratio	KD Junction Ratio
chr1:44877653|44878394	MBKD	0.434782609	0.588235294
chr10:27311487|27322306	MBKD	0.307692308	0.571428571
chr10:32740520|32762951	MBKD	0.307692308	0.615384615
chr11:33307959|33309057	MBKD	0.39408867	0.536231884
chr12:42604157|42604482	MBKD	0.235294118	0.7
chr12:100166700|100175875	MBKD	0.526315789	0.470588235
chr16:21350242|21363459	MBKD	0.666666667	0.173913043
chr17:74283273|74301022	MBKD	0.363636364	0.615384615
chr2:207144264|207162097	MBKD	0.279069767	0.506329114
chr22:25771780|25777545	MBKD	0.571428571	0.347826087
chr22:28910745|28915308	MBKD	0.352941176	0.727272727
chr3:51575514|51586079	MBKD	0.416666667	0.533333333
chr3:63898264|63898901	MBKD	0.258823529	0.522522523
chr3:149563798|149639014	MBKD	0.482084691	0.534031414
chr5:137320946|137324004	MBKD	0.43537415	0.598130841
chr7:64004085|64004810	MBKD	0.163265306	0.535211268
chr8:90734243|90737869	MBKD	0.476190476	0.666666667
chr9:4286038|4286523	MBKD	0.454545455	0.9
chr9:16727795|16738483	MBKD	0.357142857	0.684210526
chr9:98740343|98742148	MBKD	0.512820513	0.148148148
chrX:47705504|47755339	MBKD	0.388888889	0.52
chr1:224142179|224149975	TrKD	0.583333333	0.461538462
chr1:225140372|225161855	TrKD	0.533333333	0.285714286
chr11:33307959|33309057	TrKD	0.39408867	0.530973451
chr17:67270084|67280213	TrKD	0.214285714	0.689655172
chr2:148653870|148657467	TrKD	0.363636364	0.727272727
chr2:179400459|179407088	TrKD	0.631578947	0.4
chr2:207144264|207162097	TrKD	0.279069767	0.528301887
chr20:62407031|62422143	TrKD	0.724637681	0.454545455
chr3:63898264|63898901	TrKD	0.258823529	0.587155963
chr3:113077592|113085156	TrKD	0.25	0.571428571
chr3:149563798|149639014	TrKD	0.482084691	0.60625
chr4:1656705|1670632	TrKD	0.631578947	0.307692308
chr5:137320946|137324004	TrKD	0.43537415	0.56
chr9:17309056|17342442	TrKD	0.52173913	0.235294118
chrX:130826466|130928494	TrKD	0.608695652	0.25
chrX:130877124|130928494	TrKD	0.64516129	0.418604651
chr1:117944808|117984947	PTKD	0.403940887	0.588832487
chr1:247319708|247323115	PTKD	0.297619048	0.519823789
chr10:27311487|27322306	PTKD	0.307692308	0.761904762

chr10:31644073|31676727	PTKD	0.571428571	0.461538462
chr10:74468041|74475660	PTKD	0.235294118	0.761904762
chr10:126631026|126631876	PTKD	0.306666667	0.603305785
chr11:33307959|33309057	PTKD	0.39408867	0.641975309
chr11:130130751|130131824	PTKD	0.3375	0.613333333
chr12:42604157|42604482	PTKD	0.235294118	0.740740741
chr12:72051306|72054207	PTKD	0.285714286	0.588235294
chr12:111990084|111993723	PTKD	0.433179724	0.651289009
chr12:116668338|116675510	PTKD	0.144927536	0.746781116
chr12:117423011|117426674	PTKD	0.350877193	0.594594595
chr14:55168780|55169298	PTKD	0.153846154	0.571428571
chr14:99924616|99932150	PTKD	0.317647059	0.617363344
chr16:21350242|21363459	PTKD	0.666666667	0.390243902
chr16:80718435|80719026	PTKD	0.368983957	0.582608696
chr17:67270084|67280213	PTKD	0.214285714	0.545454545
chr18:9182380|9221997	PTKD	0.230769231	0.571428571
chr18:19345733|19359646	PTKD	0.327683616	0.676923077
chr18:51804073|51813781	PTKD	0.222222222	0.518518519
chr19:8995635|8997536	PTKD	0.197530864	0.503937008
chr2:45773871|45789895	PTKD	0.387755102	0.791044776
chr2:63660879|63667005	PTKD	0.318181818	0.545454545
chr2:148653870|148657467	PTKD	0.363636364	0.704225352
chr2:191523884|191537878	PTKD	0.234375	0.548672566
chr2:200233328|200298237	PTKD	0.279069767	0.72
chr2:207144264|207162097	PTKD	0.279069767	0.509803922
chr20:18278629|18287037	PTKD	0.230769231	0.551724138
chr22:24025912|24037699	PTKD	0.571428571	0.285714286
chr22:28910745|28915308	PTKD	0.352941176	0.56
chr3:63898264|63898901	PTKD	0.258823529	0.612903226
chr3:129546646|129551669	PTKD	0.380952381	0.555555556
chr3:143704385|143708679	PTKD	0.271604938	0.578947368
chr3:149563798|149639014	PTKD	0.482084691	0.720156556
chr3:157839892|157841780	PTKD	0.308943089	0.571428571
chr4:113483527|113506881	PTKD	0.235294118	0.545454545
chr4:166141086|166184511	PTKD	0.269662921	0.651162791
chr5:137320946|137324004	PTKD	0.43537415	0.701149425
chr6:47251674|47254331	PTKD	0.289855072	0.56
chr7:17908030|17937069	PTKD	0.31496063	0.504065041
chr7:23650790|23651172	PTKD	0.2	0.666666667
chr7:64004085|64004810	PTKD	0.163265306	0.547945205
chr7:99621042|99621930	PTKD	0.345454545	0.576419214
chr8:52773405|52773806	PTKD	0.372881356	0.673366834
chr8:62593527|62596747	PTKD	0.278580815	0.579124579

chr8:68030483|68049838	PTKD	0.136363636	0.530120482
chr8:131370263|131374017	PTKD	0.310679612	0.615384615
chr9:4286038|4286523	PTKD	0.454545455	0.75
chr9:16727795|16738483	PTKD	0.357142857	0.603174603
chr9:17309056|17342442	PTKD	0.52173913	0.333333333
chr9:17330630|17342442	PTKD	0.260869565	0.592592593
chr9:94479589|94484862	PTKD	0.347826087	0.558139535
chr9:97535284|97563284	PTKD	0.75	0.266666667
chr9:99658178|99665390	PTKD	0.162162162	0.592592593
chrX:47705504|47755339	PTKD	0.388888889	0.730769231
chrX:130826466|130928494	PTKD	0.608695652	0.181818182
chrX:130877124|130928494	PTKD	0.64516129	0.492753623

Supplementary Table 4. LC-switching and CTU-switching circRNAs in hepatocellular carcinoma libraries

LC-swithcing					
**circRNA_ID**	**Strand**	**Type**	**Gene_Symbol**	**Pvalue**	**Adjusted Pvalue**
chr5:72370569|72373320	+	exon	FCHO2	0.000257974	0.031563499
chr2:207144264|207162097	+	exon	ZDBF2	0.000151708	0.022556033
chr8:63845611|63846776	-	intron	NKAIN3	6.59E-05	0.013136825
chr2:120885264|120932576	+	exon	EPB41L5	2.21E-07	0.000473007
chr20:32207323|32211102	+	exon	CBFA2T2	4.41E-05	0.01131878
chr3:51575514|51586079	+	intron	RAD54L2	2.37E-05	0.008052975
chr5:137320946|137324004	-	exon	FAM13B	0.000472032	0.044056502
chr1:41536267|41541123	-	exon	SCMH1	7.37E-09	6.27E-05
chr16:80718435|80719026	-	exon	CDYL2	3.90E-05	0.010349231
chr4:88116476|88116842	-	exon	KLHL8	9.27E-05	0.016499075
chr5:135329179|135329705	+	intergenic_region		2.57E-05	0.008052975
chr7:99621042|99621930	+	exon	ZKSCAN1	1.41E-09	3.31E-05
chr4:144464662|144465125	+	exon	SMARCA5	5.08E-08	0.000217143
chr7:23650790|23651172	+	exon	CCDC126	0.000246895	0.030687527
chr12:70193989|70195501	+	exon	RAB3IP	1.69E-07	0.000417685
chr2:120885264|120932580	+	exon	EPB41L5	4.84E-06	0.003157196
chr5:67913310|67915874	-	intergenic_region		2.11E-05	0.007944582
**CTU-switching**					
**circRNA_ID**	**Strand**	**Type**	**Gene_Symbol**	**Pvalue**	**Adjusted Pvalue**
chr1:42730786|42776781	-	exon	FOXJ3	0.004702533	0.006146473
chr13:42385361|42393522	-	exon	VWA8	0.00604033	0.006146473
chr7:30590252|30614497	-	exon	AC005154.6	0.08835202	0.044952292
chr8:131164982|131193126	-	exon	ASAP1	0.000189279	0.000605329
chr2:39559058|39564722	-	exon	MAP4K3	7.84E-05	0.000438642
chr6:131247745|131277639	-	exon	EPB41L2	0.037264273	0.022546491
chr4:54292039|54310270	+	exon	FIP1L1	0.098931599	0.047122162
chr2:214174783|214239843	+	exon	SPAG16	0.011881032	0.01022984
chr3:141231005|141259451	+	exon	RASA2	0.000562904	0.001204326
chr21:38792601|38845182	+	exon	DYRK1A	0.020734594	0.014065968
chr1:146661756|146696658	-	exon	FMO5	0.014363848	0.011088194
chr1:232649603|232669329	-	intron	SIPA1L2	0.00604033	0.006146473
chr16:14687158|14698083	-	exon	PARN	0.00604033	0.006146473
chr2:165548731|165561615	-	exon	COBLL1	6.26E-06	4.67E-05
chr9:4823548|4833228	+	exon	RCL1	0.00556046	0.006146473
chr6:18160114|18166609	+	exon	KDM1B	0.00604033	0.006146473
chr14:31185130|31204064	+	exon	SCFD1	0.045316297	0.026696778
chr8:17601113|17613470	-	exon	MTUS1	6.26E-06	4.67E-05
chr2:24103509|24108699	-	exon	ATAD2B	0.056516537	0.032441354

chr1:167921038|167944253	+	exon	DCAF6	0.006557193	0.006382314
chr10:104636711|104650435	+	exon	AS3MT	0.069931212	0.038183456
chr10:93902786|93940776	-	exon	CPEB3	0.000122478	0.000456977
chr3:56694759|56707753	-	exon	FAM208A	0.093521849	0.04551382
chr6:132206071|132207864	+	exon	ENPP1	0.000115901	0.000456977
chr7:90233532|90252857	+	intron	CDK14	0.020002144	0.013993121
chr4:103446669|103459113	+	exon	NFKB1	0.013320516	0.010650035
chr5:38991051|39021238	-	exon	RICTOR	0.001348582	0.002515847
chr6:34574332|34614575	-	exon	C6orf106	0.036048327	0.022416647
chr16:48311249|48337216	+	exon	LONP2	0.02475803	0.015835657
chr3:51575514|51624575	+	exon	RAD54L2	0.000591764	0.001204326
chr6:90461150|90472249	-	exon	MDN1	0.004320184	0.006146473
chr9:37126309|37147442	+	exon	ZCCHC7	0.01929238	0.013931958
chrX:53672263|53681075	-	exon	HUWE1	0.005795918	0.006146473
chr7:102743498|102769239	-	exon	NAPEPLD	0.017938613	0.013386149
chr11:120345269|120348235	+	exon	ARHGEF12	0.023073982	0.015192585
chr10:12123471|12162266	+	exon	DHTKD1	0.000338195	0.000946379
chr8:68200190|68214701	-	exon	ARFGEF1	0.001866064	0.003213447
chr9:86354616|86383885	-	exon	GKAP1	0.07204543	0.038401235
chr5:43292576|43297268	-	exon	HMGCS1	0.00413978	0.006146473
chr2:242343243|242357524	+	exon	FARP2	2.36E-06	4.67E-05
chr15:43120126|43132631	-	exon	TTBK2	0.069931212	0.038183456
chr14:67736418|67770316	+	exon	MPP5	0.010176945	0.009113085
chr12:459787|465703	-	exon	KDM5A	0.012344746	0.010235438
chr22:28290543|28310335	-	exon	PITPNB	0.009045397	0.008437318
chr18:9583115|9595151	-	exon	PPP4R1	0.076430946	0.039791363
chr6:36492118|36507984	-	exon	STK38	0.093521849	0.04551382
chr9:123751324|123753558	-	exon	C5	0.000460073	0.001144385

Supplementary Table 5. Primers of circRNAs for qRT-PCR validation

circRNA_ID	Target	Primer	Sequence
circFIRRE	5' cirexon	forward	CAGTATGTTCTTCAAGCTGCTCT
		reverse	GCCAGGTACAGTCTTGTGTTC
	3' cirexon	forward	GATCACTAAGGTCTGTTCCCAATAC
		reverse	CCAAGTCTTCCCATTTCTACTCTTT
	BSJ	forward	GAGGAGACTAAGGTGTCAGTATGT
		reverse	GGTACAGTCTTGTGTTCTTGTAGTT
circSETD3	5' cirexon	forward	AAATGGGTAAGAAGAGTCGAGTAAA
		reverse	CTCACTGGTCAGGTTCAAGATT
	3' cirexon	forward	TGACACTCCTCTCTACTTTGAAGA
		reverse	CGTACTGTCGAGCTGTGTTT
	BSJ	forward	CCTACTTCTATAAAGTCATCCAGTCAGAA
		reverse	CTTTGGTGACACAGTTGCTGTAG
circRSRC1	5' cirexon	forward	GGACGTCGGTCATCAGATACT
		reverse	CTTTCTTTCGGCTGTATGTTCTACT
	3' cirexon	forward	CAGGCATCGATCAAGCAGTAG
		reverse	CCTCTGAACTCTATAGGATTTCCCT
	BSJ	forward	AGTAGCTCTTCTTATGGCTCCAG
		reverse	CGACGTCCCATTTCTTCTTGTT
circMIB1	5' cirexon	forward	CATGATGGAACCATGTGTGATACC
		reverse	TAATTTGTACACTCTGCACACTTCC
	3' cirexon	forward	TCATGGAGGATGGACTGATGG
		reverse	CATGATCTTCATCAATGCCACAAAC
	BSJ	forward	GGACTGATGGAATGTTTGAGACTTTA
		reverse	GCTTGATGCCTATTGCCACTT
circFNDC3B	5' cirexon	forward	CATCATCTCCCTCCCTATCTGAC
		reverse	CATATCTCCAGGTCCGGTAACA
	3' cirexon	forward	CAGTATACAATGGCTATGGGAAGG
		reverse	CTTGCTCGTCGCTCTGTTT
	BSJ	forward	GGTAGTGGTCCCGGAATTAAGA
		reverse	CCAGTACTATCTTCAATCACCTTGC
circTNP03	5' cirexon	forward	CAGGATGTGGAGTCATGCTATTT
		reverse	GTAAAGAGGCATGAGAGTCTGTG
	3' cirexon	forward	CAGTGTTACCTGAAGAAGTACATAGT
		reverse	CTGTACTAGAGTAGAAGGCCAAATC
	BSJ	forward	CGTTCCTTACGAATTGGAGCTAATC
		reverse	CCCATGCATGAACCAATAGAGATAC
hsa_intergenic_006404	BSJ	forward	GAGAAAGAGCTCAAGGCCACA
		reverse	TCTGGCTCCCTGGCTTTTATC
