# Supplementary material for: Accurate quantification of circular RNAs identifies extensive circular isoform switching events
Source: Nat Commun. 2020 Jan 3;11:90. doi: 10.1038/s41467-019-13840-9 (PMC6941955; doi:10.1038/s41467-019-13840-9)
Supplement: Supplementary file 3 — Reporting Summary [file 41467_2019_13840_MOESM3_ESM.pdf]

nature research

Corresponding author(s): Fangqing Zhao

Last updated by author(s): Nov 7, 2019

Reporting Summary

Nature Research wishes to improve the reproducibility of the work that we publish. This form provides structure for consistency and transparency in reporting. For further information on Nature Research policies, see Authors & Referees and the Editorial Policy Checklist.

Statistics

For all statistical analyses, confirm that the following items are present in the figure legend, table legend, main text, or Methods section.

n/a	Confirmed	
☐	☒	The exact sample size (*n*) for each experimental group/condition, given as a discrete number and unit of measurement
☐	☒	A statement on whether measurements were taken from distinct samples or whether the same sample was measured repeatedly
☐	☒	The statistical test(s) used AND whether they are one- or two-sided<br>*Only common tests should be described solely by name; describe more complex techniques in the Methods section.*
☒	☐	A description of all covariates tested
☐	☒	A description of any assumptions or corrections, such as tests of normality and adjustment for multiple comparisons
☐	☒	A full description of the statistical parameters including central tendency (e.g. means) or other basic estimates (e.g. regression coefficient) AND variation (e.g. standard deviation) or associated estimates of uncertainty (e.g. confidence intervals)
☒	☐	For null hypothesis testing, the test statistic (e.g. *F*, *t*, *r*) with confidence intervals, effect sizes, degrees of freedom and *P* value noted<br>*Give P values as exact values whenever suitable.*
☒	☐	For Bayesian analysis, information on the choice of priors and Markov chain Monte Carlo settings
☒	☐	For hierarchical and complex designs, identification of the appropriate level for tests and full reporting of outcomes
☐	☒	Estimates of effect sizes (e.g. Cohen's *d*, Pearson's *r*), indicating how they were calculated

Our web collection on statistics for biologists contains articles on many of the points above.

Software and code

Policy information about availability of computer code

Data collection	Public data were downloaded from Sequence Read Archive using sratoolkit (2.9.4).
Data analysis	Our proposed method CIRIquant is available at https://sourceforge.net/projects/ciri/files/CIRIquant, and simulation data was generated using CIRI_simulator (https://sourceforge.net/projects/ciri/files/CIRI_simulator.pl/download). For RNA-seq analysis, we used HISAT (2.1.0) and StringTie (1.3.3b) for quantification of gene expression levels, and MISO (0.5.4) were used for alternative splicing analysis. For Gene Ontology and KEGG pathway enrichment, we used Enrichr (http://amp.pharm.mssm.edu/Enrichr/).

For manuscripts utilizing custom algorithms or software that are central to the research but not yet described in published literature, software must be made available to editors/reviewers. We strongly encourage code deposition in a community repository (e.g. GitHub). See the Nature Research guidelines for submitting code & software for further information.

Data

Policy information about availability of data

All manuscripts must include a data availability statement. This statement should provide the following information, where applicable:

- Accession codes, unique identifiers, or web links for publicly available datasets
- A list of figures that have associated raw data
- A description of any restrictions on data availability

The sequence data generated in this study have been deposited to BIGD with the following accession number: PRJCA001600 (https://bigd.big.ac.cn/bioproject/browse/PRJCA001600).

Field-specific reporting

Please select the one below that is the best fit for your research. If you are not sure, read the appropriate sections before making your selection.

☒ Life sciences ☐ Behavioural & social sciences ☐ Ecological, evolutionary & environmental sciences

For a reference copy of the document with all sections, see nature.com/documents/nr-reporting-summary-flat.pdf

Life sciences study design

All studies must disclose on these points even when the disclosure is negative.

Sample size	We collected 26 pairs of public data which provided both RiboMinus and RiboMinus & RNase R treated libraries in the same time, and generated additional 8 pairs of samples, which provided sufficient sample size for analyses of effect of RNase R treatment. For differential expression analysis with biological replicates, we used 40 samples from previous studies, which should have enough power for statistical analysis.
Data exclusions	No data were excluded from the analyses.
Replication	For the RNA-seq data we used for assessment of RNase R treatment efficiency, we divided total RNA into three equal fractions after rRNA depletion, and construct a riboMinus and two experimental replicates of riboMinus & RNase R treated libraries.
Randomization	Randomization was not relevant to our study, as we used public datasets from previous studies, where groups were assigned according to the source of sample.
Blinding	Blinding was not necessary for the same reasons as above.

Reporting for specific materials, systems and methods

We require information from authors about some types of materials, experimental systems and methods used in many studies. Here, indicate whether each material, system or method listed is relevant to your study. If you are not sure if a list item applies to your research, read the appropriate section before selecting a response.

Materials & experimental systems

n/a	Involved in the study	
☒	☐	Antibodies
☐	☒	Eukaryotic cell lines
☒	☐	Palaeontology
☒	☐	Animals and other organisms
☒	☐	Human research participants
☒	☐	Clinical data

Methods

n/a	Involved in the study	
☒	☐	ChIP-seq
☒	☐	Flow cytometry
☒	☐	MRI-based neuroimaging

Eukaryotic cell lines

Policy information about cell lines

Cell line source(s)	HeLa (ATCC)
Authentication	None of the cell lines used were authenticated.
Mycoplasma contamination	All cell lines tested negative for mycoplasma contamination.
Commonly misidentified lines (See ICLAC register)	No commonly misidentified cell lines were used in this study.
